# Standardized treatment planning methodology for passively scattered proton craniospinal irradiation

**DOI:** 10.1186/1748-717X-8-32

**Published:** 2013-02-03

**Authors:** Annelise Giebeler, Wayne D Newhauser, Richard A Amos, Anita Mahajan, Kenneth Homann, Rebecca M Howell

**Affiliations:** 1 Department of Radiation Physics, The University of Texas MD Anderson Cancer Center, Houston, TX, USA; 2 The University of Texas Graduate School of Biomedical Sciences at Houston, Houston, TX, USA; 3 Department of Radiation Oncology, The University of Texas MD Anderson Cancer Center, Houston, TX, USA; 4 Current Address: Scripps Proton Therapy Center, San Diego, CA, USA; 5 Current Address: Department of Physics and Astronomy, Louisiana State University, Baton Rouge, LA, USA

**Keywords:** Proton, Craniospinal irradiation, CSI, Medulloblastoma

## Abstract

**Background:**

As the number of proton therapy centers increases, so does the need for studies which compare proton treatments between institutions and with photon therapy. However, results of such studies are highly dependent on target volume definition and treatment planning techniques. Thus, standardized methods of treatment planning are needed, particularly for proton treatment planning, in which special consideration is paid to the depth and sharp distal fall-off of the proton distribution. This study presents and evaluates a standardized method of proton treatment planning for craniospinal irradiation (CSI).

**Methods:**

We applied our institution’s planning methodology for proton CSI, at the time of the study, to an anatomically diverse population of 18 pediatric patients. We evaluated our dosimetric results for the population as a whole and for the two subgroups having two different age-specific target volumes using the minimum, maximum, and mean dose values in 10 organs (i.e., the spinal cord, brain, eyes, lenses, esophagus, lungs, kidneys, thyroid, heart, and liver). We also report isodose distributions and dose-volume histograms (DVH) for 2 representative patients. Additionally we report population-averaged DVHs for various organs.

**Results:**

The planning methodology here describes various techniques used to achieve normal tissue sparing. In particular, we found pronounced dose reductions in three radiosensitive organs (i.e., eyes, esophagus, and thyroid) which were identified for optimization. Mean doses to the thyroid, eyes, and esophagus were 0.2%, 69% and 0.2%, respectively, of the prescribed dose. In four organs not specifically identified for optimization (i.e., lungs, liver, kidneys, and heart) we found that organs lateral to the treatment field (lungs and kidneys) received relatively low mean doses (less than 8% of the prescribed dose), whereas the heart and liver, organs distal to the treatment field, received less than 1% of the prescribed dose.

**Conclusions:**

This study described and evaluated a standardized method for proton treatment planning for CSI. Overall, the standardized planning methodology yielded consistently high quality treatment plans and perhaps most importantly, it did so for an anatomically diverse patient population.

## Background

The number of cancer centers that offer proton therapy as a treatment option is rising. Within the last 10 years, the number of proton centers worldwide has almost doubled; from 22 in 2003 to 38 in 2012. The rate of increase has been even more pronounced in the United States: in 2003, there were 3 active centers in the U.S., and currently there are 10 active centers, with 6 more scheduled to open within the next 2 years. As a result, there is increased interest in comparing treatment efficacies and toxicities from proton therapy to those of photon therapy [1-3]. This is especially true for children, [4-6] for whom survival rates are high, making proton therapy’s potential for sparing normal tissues particularly relevant. Particular attention has been paid to craniospinal irradiation (CSI) in pediatric patients [7-10] because: (1) CSI is a standard component in the treatment for medulloblastoma, the second most common solid tumor in children [11-13]; (2) the potential to reduce dose anterior to the spine with proton CSI could lead to reduced toxicity in organs such as the thyroid, heart, and lungs [13,14]; and (3) still-developing normal tissues in children are highly radiosensitive [15]. As a result, treatments that irradiate large portions of the body, such as CSI, are of particular concern.

Until recently, the limited number of proton centers in operation has limited the amount of patient-outcomes data available for review, especially for pediatric patients. As a result, comparisons between proton and photon treatments have typically been performed using indirect comparisons using case studies or nonrandomized groups [2,16]. Additionally, many previous studies have been criticized for focusing on improvements in proton therapy (i.e., comparing outcomes between different proton techniques) rather than comparisons between proton and photon therapies [1,3,16-19]. Thus, although the existing studies provide valuable insight, there is a definitive need for studies that rigorously compare the risks and benefits of using proton and photon irradiation in clinical settings. Because the results of such studies are highly dependent on the target volume definition and treatment planning techniques, standardized, i.e., documented and reproducible, methods of proton and photon treatment planning are needed to reliably perform these comparisons across a population of patients. Treatment planning for photon CSI is well understood and thoroughly described in the literature [20-22]. Conversely, a comprehensive description of treatment planning for proton CSI does not exist in the literature.

The objective of this study was to describe and evaluate a standardized method for proton treatment planning for CSI. We present a detailed methodological description of the planning techniques used at our institution, at the time of this study, for proton CSI. Then, we describe application of this methodology to a sample population of 18 pediatric patients. Finally, we compared dosimetric properties of the treatment plans resulting from the methodology to determine whether the methodology yields consistently high quality plans.

## Methods

### Facility-specific technical information

To better enable comparison between the treatment techniques described here and those used by other institutions, we briefly review the technical features our treatment system, i.e., The University of Texas M.D. Anderson Cancer Center Proton Therapy Center Houston (MDACC-PTCH), that are of relevance to passively scattered proton CSI. The proton treatment system (Probeat, Hitachi America Limited, Inc, Tarrytown, USA) consists of a synchrotron (70–250 MeV) and a beam transport system that bifurcates into six treatment stations. The CSI treatments described in this work were planned for rotating gantries with passive-scattering nozzles; we have two such gantries at our institution, allowing patients to be easily switched between treatment rooms. Specifically the nozzle includes a rotating range modulator wheel, a binary stack of range shifters, a contoured and compensated double scattering system, beam monitoring instrumentation, collimators, and a range compensator. This treatment equipment can produce beam energies (defined at the nozzle entrance) of 100, 120, 140, 160, 180, 200, 225, and 250 MeV, spread-out-Bragg-peaks ranging in size from 2 to 16 g/cm^2^, and field sizes of up to 25 x 25 cm^2^. The dose rate depends on the scatterer (small, medium, or large) that is in place. CSI fields are commonly treated with a large scatterer and have a dose rate of approximately 1 Gray (Gy) min per minute at isocenter. The layout, capabilities, and performance characteristics of the treatment equipment were previously described in detail in the literature [23,24].

The various modes proton beam operation (treatment, physics, and service) is managed by an overall control system. In addition, there is an independent safety control system. Data management is handled by the oncology information system (MOSAIQ, IMPAC Medical Systems Inc Sunnyvale, CA) [24]. For proton treatment planning, we use a commercial treatment planning system (TPS) (Eclipse version 8.9, Varian Medical Systems, Palo Alto, CA). Dose calculations are performed with a proton pencil beam algorithm [25] on a 2.5-mm isotropic dose calculation grid.

The patient treatment couch (in the treatment room described above) is a computer controlled robotic system with three orthogonal axes of translation (vertical, longitudinal, and lateral) and three orthogonal rotational axes (pitch, roll, and yaw). Because of the large size of the gantry and its pit, the couch base is mounted outside of the gantry and the couch top is mounted to the couch base in a cantilevered configuration. The couch top extension, the portion of the couch top in contact with the patient, is predominantly made of a carbon fiber composite material that was specially selected for its strength, uniform transparency to the beams of kilovoltage photons used for radiographic patient positioning and alignment, and uniform and minimal perturbation of proton treatment beams.

### General plan overview

The beam arrangements for proton CSI include two opposed lateral oblique cranial fields and postero-anterior spinal field(s). For standard-risk medulloblastoma patients, we prescribe 23.4 Gy (RBE [relative biological effectiveness]) (i.e., 21.3 Gy × 1.1 RBE). The units of Gy (RBE) are assigned in accordance with our clinic’s standard of care and the recommendations of ICRU Report 78, [26] which assumes proton beams have a higher RBE than photon beams. The fractionation schedule is 1.8 Gy per fraction for 13 fractions with 3 junction shifts. Typically, junction shifts are 1–2 cm apart, depending on spine length. The general clinical guidelines used for proton CSI included the following: (1) coverage of the cerebrospinal fluid (CSF) by the 100% isodose to lower end of the thecal sac (S2 or S3); (2) good coverage of the anterior skull base; (3) as much coverage as possible to the cribriform plate, balanced with planning criteria that the 100% isodose does not intercept the eyes; (4) maximum isodose line intercepting the thyroid should be 5% or less (isodose lines visually evaluated slice by slice); (5) no overshoot into the esophagus; and (6) a homogeneous dose across the the spinal cord without excessive hot or cold spots (> 105% of the prescribed dose or < 95% of the prescribed dose).

### Patient immobilization and imaging

**Figure 1:**
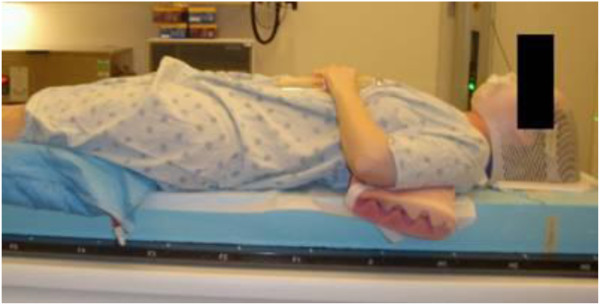
Photograph of a patient in the set-up position for proton CSI.

**Figure 2:**
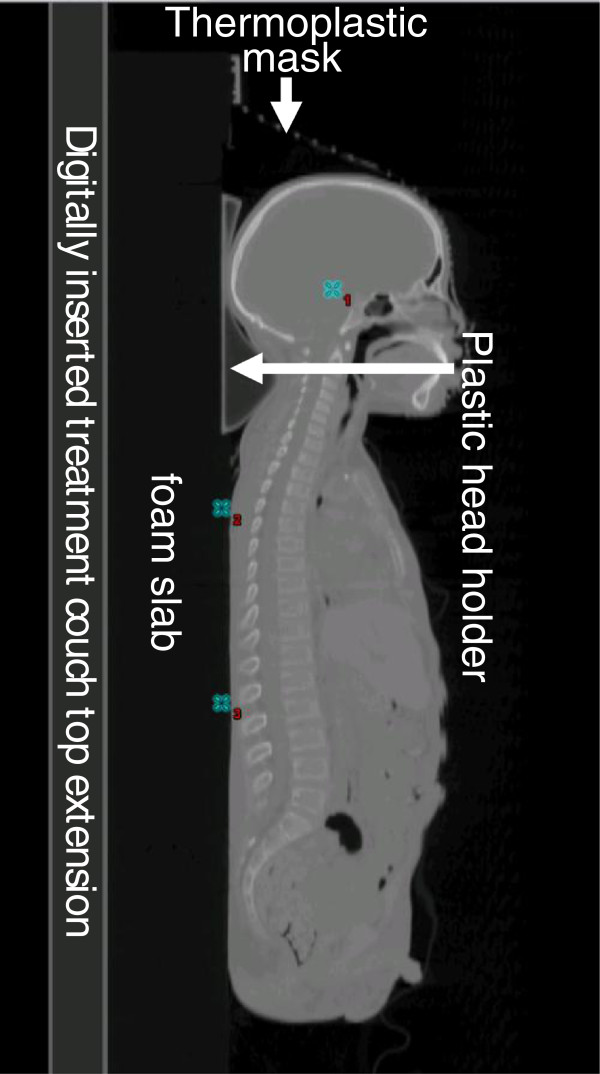
**Sagittal CT image showing the patient set-up and immobilization for representative patient (patient 10: male, age 9).** The window and level of the image was adjusted to visualize the digitally inserted couch top extension (the portion of the couch that is in contact with the patient), foam pad, thermoplastic mask, and plastic head holder. Isocenters for the cranial field(s), upper spine filed, and lower spine field are indicated with blue **×** and labeled 1, 2, and 3 respectively.

The immobilization technique used at our institution for proton CSI is described below; it has, in our experience, led to reproducible set-ups. Patients undergo computer tomography (CT) simulation in the supine position on a (10 cm thick) polystyrene foam slab (Associated Foam Plastics, Houston, USA) that is inserted between the patient and the table top. The foam slab is a rigid hard plate and does not conform to the patient. This slab elevates the patient so that oblique cranial fields do not pass through the treatment couch. Additionally, the patient’s head is immobilized with a thermoplastic mask (WFR/Aquaplast Corp. and Qfix Systems, LLC, Avondale, USA) and plastic head holder (Medtech, CIVCO, Orange City, USA) to optimize the neck curvature for patient sedation needs and cranial field placement. A photograph and a sagittal CT image of the patient set-up and immobilization are show in Figures 1 and 2, respectively.

After the patients are immobilized, CT images are acquired using a multi-slice CT scanner (General Electric LightSpeed RT16, GE Healthcare, Waukesha, USA). CT images are acquired with a 2.5-mm slice thickness in an area that extends approximately 2–3 cm superior to the patient’s thermoplastic mask holder to approximately 3–4 cm inferior to the patient's sacrum. Additionally, the entire thickness of any object with potential to be in the beam path is included in the scan. Intravenous contrast agents are not used in CT imaging for CSI planning because currently available agents do not enhance our ability to delineate the CSF target. In addition, use of such agents would artificially increase the Hounsfield units (HU) values, which could result in overshoot of the proton beams, i.e., irradiation of tissue distal to the craniospinal axis [27].

### Image processing and contours

Before the CT scan is imported into the TPS, it is post processed to delete the portion of the image containing the CT couch top extension (the portion of the couch that is in contact with the patient, Figure 2) and to insert a digital representation of the extension in its place. Inserting a treatment couch top that mimics the physical dimensions and materials of the actual treatment couch top is important because the accuracy of the delivered proton beam range requires accurate knowledge of the materials through which the beam passes during the planning process. The digital couch used at our institution was described elsewhere [28] and a similar digital couch was reported for treatment planning at the proton facility in Korea [29].

Similar to conventional photon therapy, target volume contours in proton therapy are used to define the lateral shape of the treatment field (i.e., block definition). However, unlike their photon counterparts, contours for targets and critical structures in proton treatments are used by the TPS to automatically select beam range in the patient and the width of the spread-out Bragg peak (SOBP) so that it covers the target in the direction of the beam axis. To insure that the TPS correctly assigns these machine parameters, the HU are manually reassigned for any contours that include high density objects or imaging artifacts. Surgical clips or screws, and catheters are examples where HU are re-assigned for CSI treatment planning; such manual corrections were required for about one-third of the patients included in this study. These manual heterogeneity corrections are particularly important for spinal treatment fields that are oriented in the posterior-anterior direction, with the spinal target in the posterior aspect of the patient and most normal tissues at risk are either anterior or lateral to it. Heterogeneity correction methods for proton treatment planning were described in detail elsewhere [30].

**Figure 3:**
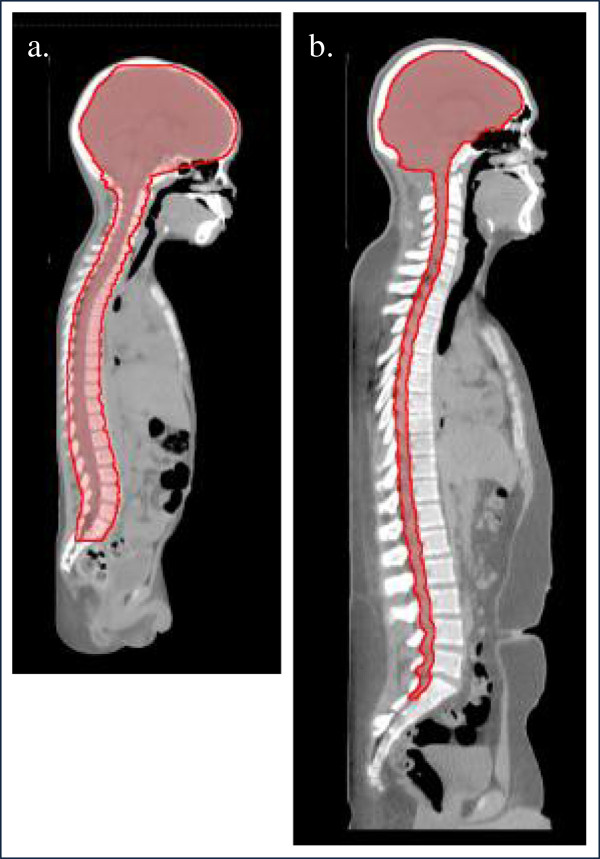
**Age-specific target volumes for two patients.** (**a**) Target volume for a 4-year-old patient included the spinal canal and the entire vertebral body, and (**b**) target volume for a 15-year-old patient included only the spinal canal. Both volumes also included the brain.

For all proton CSI patients, the CTV contour includes the entire CSF space (including the brain, and spinal canal through the cauda equina to the level of the S2/S3 vertebral junction [Figure 3b]). However, to account for some of the age-specific considerations of designing spinal treatment fields, we define the anterior edge of the target volume in the spine in two ways (Figures 3a, b). For patients who have not reached skeletal maturity as determined by bone age, (typically those younger than 15 years of age), the target volume includes the spinal canal and an additional normal tissue target volume (NTTV), which includes the entire vertebral bodies. As we described in a recent study [31], the rationale for this “is to avoid sharp dose gradients in the vertebral bodies in patients whose skeletons are still maturing. More specifically, proton treatments that are designed to irradiate only the spinal canal have high dose gradients distal to the spinal canal and lead to non-uniform irradiation of the vertebral bodies. Uniformly irradiating a larger target volume that fully encompasses the vertebral bodies is thought to reduce the risk of asymmetric growth of the vertebral body in patients whose skeletons are still maturing, [7,32], i.e., those under the age of 15 years.” For patients older than 15 years, the spinal portion of the CTV includes only the spinal canal and extends no more than 2–3 mm into the vertebral bodies, reducing the dose to bone marrow which may allow for better tolerance of the chemotherapy that is required.

### Field-specific details

Cranial fields in pediatric proton CSI are initially defined by a target volume that includes the brain contour and portions of the upper spine contour. The cranial fields are typically angled 15 degrees from the horizontal plane to reduce dose to the lens and improve dosimetric coverage of the cribiform plate. Collimators are generally not rotated, and a sufficient air gap (approximately 12–15 cm) is provided to avoid collisions of the nozzle and couch top. When designing the cranial fields, the inferior field edges are typically designed first. They extend to the patient’s shoulders to allow for feathering of the cranial-spinal field junctions, i.e., the edges of the cranial fields in the remaining junction-shifted plans are approximately 1 cm and 2 cm superior to the shoulders. The remaining field edges are defined superiorly by adding approximately 2.5 cm to the superior aspect of the brain contour (for flash), and laterally by extending the brain contour inferiorly to the shoulders with a 2.5 cm margin on the patient’s neck and editing the anterior neck contour off the oral pharynx and mandible as much as possible. Additionally, the right and left cranial fields are individually edited so that their respective field edges approximately bisect the right and left ocular globes, do not include either lens, and maintain as much coverage of the cribriform plate as possible.

**Figure 4:**
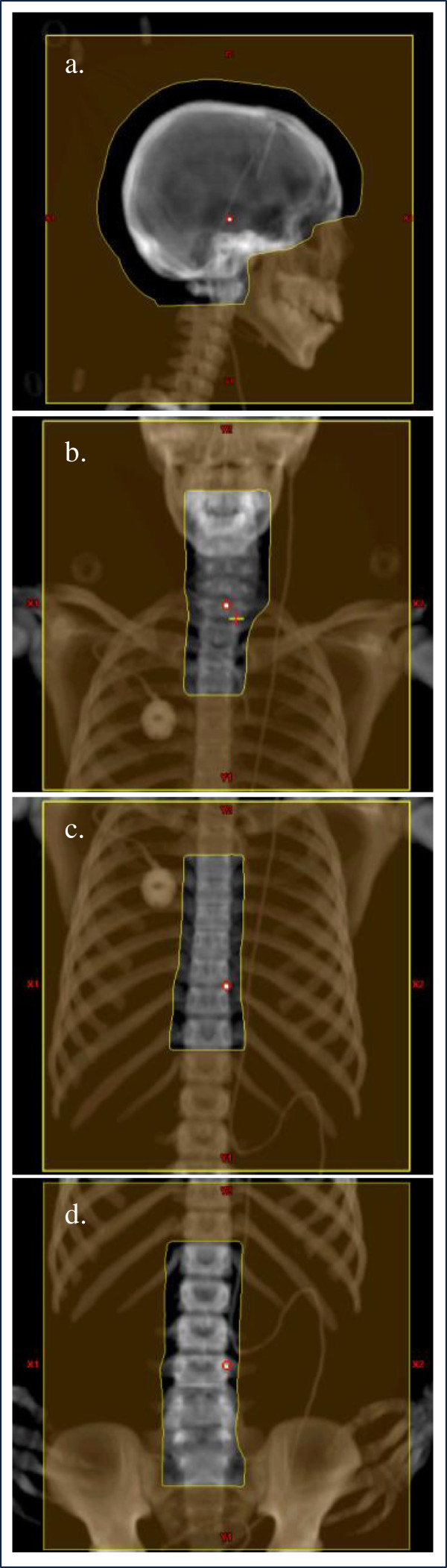
**Proton therapy cranial and spinal field borders for CSI (a representative patient) representative patient) in the (a) cranial field, (b) upper spinal field, (c) middle spinal field, and (d) lower spinal field.** Field isocenters are indicated with red circle.

The spinal fields are initially defined by the CTV and margins that include allowances for uncertainties in the depth and lateral directions. The number of spinal fields varies on the basis of spine length. In all cases, the superior border of the uppermost spinal field is matched to the inferior border of the cranial fields. Then, depending on the length of the spine, a second spinal field is matched to the inferior border of the upper spinal field, and if needed, a third spinal field is matched to the inferior border of the second spinal field. Lateral field edges are defined using a 1-cm margin around the spinal target volume. Typical field borders for a representative adolescent patient are shown in Figure 4.

Cranial and spinal field isocenters are selected during treatment planning and are defined to simplify patient setup. The field isocenters for the cranial fields are placed at the patients’ midline such that they are, at minimum, 3 cm from the field edges, i.e., 3 cm from the internal edges of the cranial blocks. The field isocenter for each spinal field is located at the same x coordinate (left-right axis) as the cranial fields. The isocenter’s y coordinate (anterior-posterior axis) is fixed for all spinal fields but differs from that of the cranial fields; it is set in the polystyrene foam pad posterior to the patient such that the couch height in the room is high enough to reduce collision issues. The z (superior-inferior axis) coordinate differs for each spinal field and is placed midway between each field’s superior and inferior borders**.** Typical field isocenters for a representative adolescent patient are shown in Figures 2 and 4.

### Uncertainty margins

Uncertainty margins are designed for each treatment field to ensure coverage of age specific target volumes (described in the following section). Uncertainty margins are calculated for each treatment field using a methodology similar to that used in our previous studies [30,33] and following the methods originally outlined by Moyers and Miller [34] and Moyers et al. [35]. The distal uncertainty margin (*DM*) is determined using.

(1) $$\mathrm{DM}=\left(0.035\times C_{d}\right)+0.3\mathrm{cm}\text{,}$$

where 3.5% of *C*_*d*_, the water-equivalent depth (cm) to the distal edge of a field’s target contour, is used to account for uncertainties in the CT number and the conversion of the electron densities to proton relative linear stopping powers. The 0.3 cm is added to account for range uncertainty due to variations in accelerator energy, material thickness in the scattering system, and compensator density.

The proximal uncertainty margin, *PM*, is determined using

(2) $$\mathrm{PM}=\left(0.035\times C_{p}\right)+0.3\mathrm{cm}\text{,}$$

where *C*_p_ is the water-equivalent depth (cm) to the proximal edge of the field’s target contour. The lateral uncertainty margin, *LM*, is determined using

(3) LM=IM+SM+P

where *IM* represents the margin for internal motion (cm), *SM* represents setup uncertainty (cm), and *P* represents the 50%–90% penumbral width (cm). Thus, the value of *LM* defines the lateral expansion of the field-specific apertures beyond the cranial and spinal target volumes. According to our standard of care, *IM* is taken to be 0 cm and *SM* 0.3 cm*.* For the spinal fields, this typically results in an *LM* value of 1 cm*. LM* is also considered for the cranial fields, but it serves as a baseline on which flash is added to the superior aspect of the treatment fields.

Although the uncertainty margins for the cranial and spinal fields are determined using the same equations, there are some differences in how the margins are implemented. For the cranial fields, *PM* and *DM* values are applied as planning parameters. That is, the TPS selects the beam range, modulation, and energy based on the requirement that the prescribed dose is delivered to the cranial target and its margins. Conversely, because there is a one-to-one correspondence between each spinal target and spinal field, values of *PM* and *DM* are incorporated into the spinal targets, which expands the target region proximal and distal to the spinal canal. This approach streamlines the optimization process because it provides visualization of the uncertainty margins relative to the dose distribution. As a result, edits for optimization can be made judiciously.

Once the uncertainty margins are determined, the following equation based on the works of Moyers et al. [35] and Urie et al. [36] is applied. This equation defines the amount of expansion or smearing of the range compensator. The equation effectively decreases the range compensator thickness such that proton penetration depth in the patient is increased in order to maintain distal coverage in the presence of radiological path length variations due to internal anatomical motion; note that this correction is variable across the compensator and is not a single fixed value. It is defined using

(4) $$S=\sqrt{{\left(\mathrm{IM}+\mathrm{SM}\right)}^{2}+{\left(C_{d}\times 0.03\right)}^{2}}\text{,}$$

where *S* is the amount of smear determined using the quantities *IM, SM*, and 3% of the *C*_d_. *S* is calculated for the spinal fields only. Most cranial fields are not designed to have range compensators because even in the case of maximum smear, the milling structure of the compensator does not match the curvature or thickness gradients of the patients’ skulls. Note that our comment rereading not using range compensators for most brain fields specifically refers to the type of cranial fields which are intended to cover the whole brain; range compensators are used when planning boost fields (not discussed in this study) because the field is specifically conformed to cover a particular gross target volume rather than the entire brain).

### Optimization of dose distributions

After the initial fields are optimized and calculated, the dose distributions are reviewed, and we begin the process of field-specific optimization. That is, each field is individually optimized to provide uniform coverage of target volumes and meet constraints on dose to normal tissues. Typically, optimization begins with the cranial fields. Cranial field optimization is done in several steps. First, the general shape of the 23.4 Gy (RBE) isodose line is reviewed to ensure that it includes the cerebrospinal fluid in the subarachnoid space. Ideally, this means that the 23.4 Gy (RBE) isodose line extends minimally to the interior edge of the skull and maximally 1–2 mm outside the exterior edge of the skull. If needed, adjustments to the general shape of the 23.4 Gy (RBE) isodose line are made through changes to the proximal and distal margins of the cranial fields. Then, to improve dosimetric homogeneity within the cranial target region, we edit the plan’s dose normalization value by selecting an initial normalization value to create uniform coverage of the cranial target region by the 23.4 Gy (RBE) isodose line. The normalization is finely adjusted to reduce the higher dose values or hot spots (at least 24.6 Gy (RBE)) that would result from scatter in immobilization devices and variations in skull thickness. At this point, if they were being used, range compensators would be manually edited to attenuate dose to the cochlea and smoothed (further smeared) to reduce their impact on dosimetric inhomogeneity in the brain. For plans prescribed to 23.4 Gy (RBE), most compensators are removed to reduce the number of hot and cold streaks (> 105% of the prescribed dose or < 95% of the prescribed dose) in the cranial dose distributions. For plans prescribed to 36 Gy (RBE), use of range compensators to reduce dose to the cochlea is evaluated on a case-by-case basis.

Next, the spinal fields are optimized for uniform coverage of the 23.4 Gy (RBE) isodose line relative to the age-specific spinal target. This process begins with the uppermost spinal field and ends with the lowest spinal field and involves several steps. First, the general shape of the 23.4 Gy (RBE) isodose line is assessed relative to the age-specific spinal target. Ideally, the distal edge of the 23.4 Gy (RBE) isodose line coincides with the distal edge of the spinal target, and the proximal edge of the 23.4 Gy (RBE) isodose line coincides with the proximal edge of the spinal target or, maximally, just anterior to the dorsal skin surface. If needed, the general shape of the 23.4 Gy (RBE) isodose line is adjusted relative to the spinal target by making small changes to the proximal and distal margins of the spinal fields. Second, to improve dosimetric uniformity within the spinal target region, the weight of the spinal field is adjusted relative to the plan normalization value. In general, spinal field weights can differ from their initial setting of 1.0 by ±0.5% to ±5%, and in most cases, the largest weight is assigned to the field incident upon the sacrum. Third, field-specific range compensators are edited to reduce hot and cold spots in the dose distribution. These edits differ slightly for each field because of differences in anatomy. In the upper spinal field, edits are made to reduce hot and cold spots due to scatter from the head holder or changes in the neck curvature (e.g., interfraction variation in neck flexion). Additionally, compensator thickness is increased in the thyroid region to block isodose lines that are greater than 5% of the prescribed dose from crossing the medial edge of the thyroid contour. In the spinal field incident to the sacrum, edits are made to increase compensator thickness on the basis of the patient’s sacral curvature. In all cases, field-specific edits to the compensators are made and then smoothed (i.e., applied a smoothing function to remove any abrupt steps in compensator topography which can occur when a compensator is manually edited) to ensure that small shifts in the patient setup do not compromise dosimetric coverage of the spinal canal.

After, the spinal fields are optimized for uniform dosimetric coverage of the age-specific spinal target, attention is focused on reducing dose inhomogeneities at the field junctions. The junction region is defined as the region where the edges of the cranial/spinal and spinal/spinal fields align: 2–3 CT slices in the superior direction and 2–3 CT slices in the inferior direction. This region is 1.25-1.50 cm in length, is contained laterally and distally within the spinal target and is shifted on a weekly basis. In the junction region, hot spots are defined by isodose lines greater than 108% of the prescribed dose, and cold spots are defined by isodose lines less than 95% of the prescribed dose. Hot spots and cold spots are removed using three procedures: edits to the apertures forming the field junction, fine edits to the field weights, and edits to the abutting fields' compensators. Edits to the apertures are made so that the field edges will be precisely aligned to one another. Once the fields are precisely aligned, the field weights are finely adjusted to balance coverage of the spinal canal with the 100% isodose line and reductions in the dose at the field junctions. After the field weights are optimized, remaining regions of isodose lines greater than 110% of the prescribed dose may indicate fine adjustments to the field apertures are needed to improve field alignment. After that, the field compensators are edited, if needed. These edits are designed to reduce hot spots in the spinal canal by removing steep gradients within the compensator and shifting hot spots from the spinal canal to the vertebral body.

Finally, to assess the combined effects of the optimization processes, the display of cranial and spinal target volumes are turned off, and the location of the 100% isodose line relative to the interior border of the skull and spinal canal is evaluated for each of the three junction-shifted plans. If needed, final edits to the isodose coverage are made by scaling the 100% isodose line in an individual plan, but because this final edit scales the dose distribution for all treatment fields in an individual plan, it is rarely used. Once all three junction-shifted plans individually meet the planning criteria, they are combined and re-evaluated as a summed plan. Then, based on the dosimetric evaluation of the summed plan, final edits to the individual junction-shifted plans are performed, if needed, until the summed plan meets all objectives and constraints.

### Patient population

To evaluate the quality of our treatment planning methodology, we applied the methodology to an anatomically diverse and representative population of patients. Eighteen pediatric patients (ages 2 to 16 years of age) were selected using the consecutive sampling method [37]. The inclusion criteria were male and female patients between 2 and 18 years of age at the time of treatment who received proton CSI between 2006 and 2009 at the UTMDACC-PTCH. Patients who received photon therapy or who were not simulated in the supine position were excluded. The sample was fairly evenly distributed in age and sex. The patients varied with respect to anatomical stature, age, and the corresponding volumes of their internal organs (Table 1). For each patient the same planning process was employed.

**Table 1:** Patient demographics including volumes of organs of interest, for which each organ was contoured in its entirety^*^

**Index**	**Sex**	**Age**	**Thyroid**	**Lungs**	**Liver**	**Kidneys**	**Heart**	**Esophagus**	**Eyes**	**Lens**	**Spinal Cord**	**Brain**
		**(y)**	**(cm3)**	**(cm3)**	**(cm3)**	**(cm3)**	**(cm3)**	**(cm3)**	**(cm3)**	**(cm3)**	**(cm3)**	**(cm3)**
1^€^	F	2	1.9	320.8	372	63.5	139.5	1.2	16.2	0.3	41.4	1047
2	F	4	3.2	548.1	633.8	127	218.9	8.8	14.7	0.2	53.5	1336
3	F	6	3.3	1062	631.5	125.4	158.9	4.7	16.5	0.3	49.1	1456
4	F	8	4.5	1048	733.5	136.9	258.6	6.9	14.1	0.2	32.5	1402
5	F	9	3.8	1072	886.4	190.9	264	8.6	18.9	0.3	47.9	1318
6	M	3	1.5	457.9	595.9	137.8	96.6	5.5	12.3	0.1	19.5	1554
7	M	4	3.9	626.6	596	110	199.8	15.1	25.3	0.4	56.4	1380
8	M	6	4.1	814.8	847.4	160.8	214.5	5.3	16.6	0.2	83	1481
9	M	8	2.3	881.4	556.9	135.1	222.9	6.9	11.9	0.2	69.5	1471
10	M	9	5.4	1116	1066	117.4	276.9	7	10.8	0.2	48.4	1253
11	F	12	1.7	1479	646.4	169.4	243.1	8.7	12.8	0.2	26.9	1204
12	F	13	4.3	796.1	1429	198.1	227	13.6	14.9	0.1	32.5	1266
13^*^	F	16	7.7	1850	1867	283.5	474.6	18.9	15.8	0.3	82.1	1279
14	M	12	11.7	1673	1354	287.2	434.2	15	14.2	0.1	111.1	1335
15^*†^	M	13	12.6	2482	1498	342.5	417.4	18.6	16.5	0.2	42.3	1907
16	M	14	7.8	1247	1006	207.3	53.7	10.3	22.2	0.2	93.3	1533
17^*^	M	15	10	2273	1816	356.6	493.1	18.3	15.2	0.1	110.9	1401
18^*^	M	16	14.3	2308	1869	487.1	821.9	24	19.4	0.4	169.2	1542
Mean (cm3)		9.4	5.8	1225.3	1022.5	202.0	289.8	11.0	16.0	0.2	65.0	1397.8
SD (cm3)		4.5	3.9	636.5	479.9	105.6	176.3	6.0	3.5	0.1	36.7	177.3

^*^ Indicates patients whose target volume included the CSF and the entire vertebral bodies. All other patients’ target volumes included only the CSF.

^†^ Patient was 13 years of age at the time of treatment, but assumed to be at skeletal maturity for treatment planning.

^**€**^ Due poor contour resolution, volume estimates for the esophagus and thyroid of patient #1 were not optimal. However, sensitivity tests indicate that changes in the volumes for these organs by as much as 300% did not affect the mean values for the population.

### Evaluation of planning methodology

For each patient the treatment plans were evaluated to assure adherence with our clinical guidelines by reviewing the isodose distributions (on each axial CT slice) and dose volume histograms (DVH) for the ten organs of interest (lungs, liver, heart, kidneys, spinal cord, brain, eyes, lenses, esophagus, and thyroid). Two representative patients (indices 10 and 13) were selected for presentation in the results section of this manuscript. Patient 10 (age 9) is representative of the younger patients planned with the larger age-specific target volume that included CSF and vertebral bodies. Patient 13 (age 16) is representative of the older patients planned with the smaller age specific target volume that included only CSF. Both patients were of average stature and their organ volumes were within the corresponding one standard deviation of the mean organ volumes of the population.

After evaluation of individual patient isodose distributions and DVHs, we completed population-based dosimetric and statistical analyses. First, average DVHs were generated for ten organs of interest by calculating the average values of D_5%_ to D_100%_ (in 5% increments) and maximum dose (D_max_). Next, for the same ten organs, average values for minimum dose (D_min_), D_max_, were calculated separately for the entire population of patients and for the two subgroups of patients planned with the two different age-specific target volumes. Values of mean dose (D_mean_) were similarly calculated. Finally, to quantify the variation in machine parameters between patients’ treatment fields, we determined the minimum, maximum, and median for the proton beam range, energy, and SOBP width for our population’s cranial and spinal fields.

## Results

### Machine parameters for treatment delivery

Machine-specific treatment parameters for all 18 proton CSI treatment plans are summarized in Table 2. As described in Methods, these values were automatically determined by the TPS on the basis of the treatment-specific target contours. The variation in the proton beam range, energy, and SOBP width within this population is largely attributed to the wide interval in patient age and, thus, the wide interval in patient size. In general, median values for range, energy, and SOBP width were higher for the cranial fields than for the spinal fields. This difference is attributed to the larger axial size of the cranial target than of the spinal target.

**Table 2:** Values for the minimum, maximum, median, and standard deviation (SD) across the population (N = 18) for beam range (cm), energy (MeV) and width of the modulated range or spread out Bragg peak (SOBP) (cm)

		**Cranial fields**	**Spinal fields**		
			**Upper**	**Middle**	**Lower**
Range (cm)	Min - Max	14.7-20.60	8.5-15.4	6.6-13.6	7.6-16
	Median	17.0	10.9	12.3	11.1
	SD	1.3	1.9	2.7	2.2
					
Energy (MeV)	Min - Max	180-225	140-200	140-200	140-200
	Median	200	160	180	160
	SD	12	17	18	17

SOBP (cm)	Min - Max	15-16	4-7	4-8	4-7
	Median	16	5	5	6
	SD	1	1	2	1

Energy values correspond to set energy steps of 140 MeV, 160 MeV, 180 MeV, 200 MeV, and 225 MeV. Abbreviation in this table: spread out Bragg peak (SOBP).

### Dosimetric results

**Figure 5:**
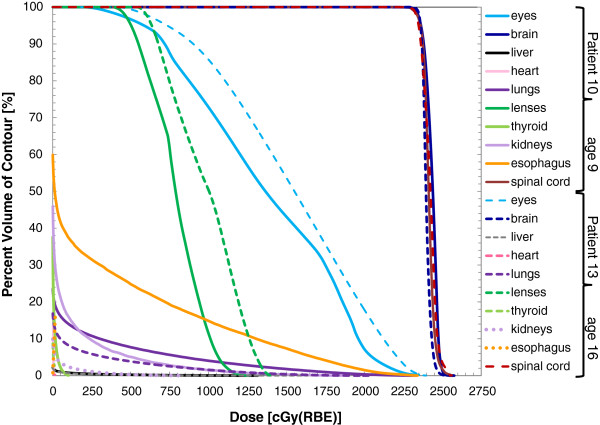
**Dose volume histograms (DVH) for two representative patients with different age specific target volumes.** Solid lines are for patient 10: male, age 9 and dashed lines are for patient 13: female, age 16.

**Figure 6:**
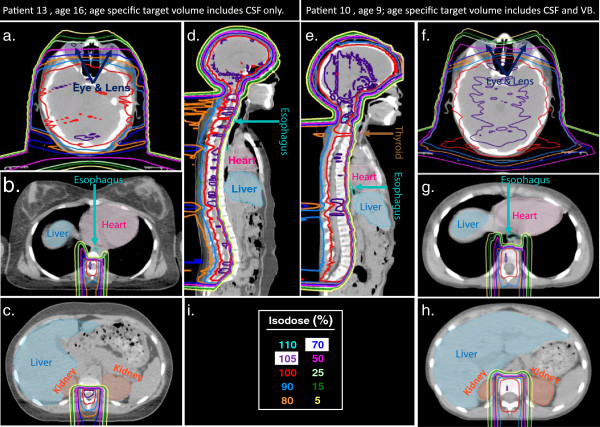
**Dose distributions for two representative patients with different age specific target volumes.** Panels on the left are for patient 13, female, age 16 and panels on right are for patient 10: male, age 9. (**a,e**) Dose distribution in the axial plan at the level of the eyes. (**b,g**) Dose distribution in the axial plane, taken at the vertebral space between T8-T9 to show dose relative to the liver, heart, lungs, esophagus, and cord. (**c,h**) Dose distribution in the axial plane, taken at the vertebral space between L1-L2 to show dose relative to the liver, kidneys, and cord. (**d,e**) Dose distribution in the sagittal plane, taken at a poisiton lateral to the cord to include aspects of the thyroid and esophagus in the image. (**i**) Isodose scale for images (**a–h**).

The DVHs and isodose distributions for two patients (patient 10 and 13) are shown in Figures 5 and 6, respectively. These plans were was optimized to deliver mean doses between 103% and 104% of the prescribed dose to the spinal cord and brain while dose to the eyes, esophagus, and thyroid were reduced. Organs such as the lungs, kidneys, heart, and liver were not specifically identified for dose reduction, but using the outlined methodology, dose to these organs was minimized. The data for these two patients was typical of our observations for the entire population of patients. That is that good normal tissue sparing was achieved for all patients but normal tissue sparing was better in the older patients when compared to the younger patients.

**Figure 7:**
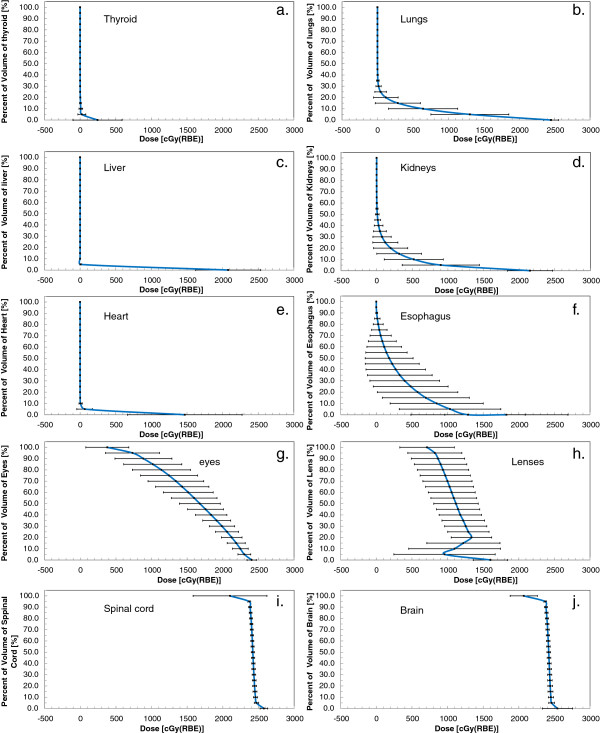
**Average dose volume histogram (DVH) for the 18 patients in this study.** Plots were generated by calculating the mean values of D_5%_ to D_100%_ (in 5% increments) and D_max_ for each organ contour. The error bars indicate one standard deviation of the mean. (**a**) thyroid, (**b**) lungs, (**c**) liver, (**d**) kidneys, (**e**) heart, (**f**) esophagus, (**g**) eyes, (**h**) lenses, (**i**) spinal cord, and (**j**) brain.

Organ-specific, population-averaged DVHs (for the 18 patients) are shown in Figure 7 for ten organs. In Table 3, population-averaged dose-volume results (*D*_min_, *D*_max_, and *D*_mean_) for ten organs are reported separately for the entire patient population, and for the two subgroups of patients, they are stratified according to the age-specific target volumes. Overall, the standard deviations in the organ doses were lower for the subgroups compared to the population as a whole (Table 3). The brain and spinal cord were part of the target volume and naturally had high doses for all patients, with little difference in D_min_, D_max_, and D_mean_ observed between the entire population and subgroups. Additionally, our planning technique produced similar results across the population of patents considered, as evidenced by low standard deviations in the D_min_, D_max_, and D_mean_ values (Table 3) and the D_5%_ through the D_95%_ values (Figure 7) for the brain and spinal cord. For the remaining eight tissues considered, good normal tissue sparing was achieved for all of the patients (Table 3, Figure 7). However, compared to the brain and spinal cord, there was larger standard deviation in doses. This standard deviation was largely related the age specific target volumes and to the proximity of the target volume to the organ. The eyes and the lenses received higher doses than other organs. They are close to the cranial fields, and even with optimization, dose could not be entirely eliminated by editing field-defining apertures because of the finite penumbral width. Not surprisingly, the dose to the eyes and brain were similar in all patients, including the patients with different age-specific spinal target volumes because the cranial component of the target volume was the same in all patients. The kidneys and lungs are both lateral to the spinal fields, and therefore dose in these organs was dependent on the lateral margins assigned to the spinal target volume, which varied with patient age and size. In the kidneys and lungs, higher doses were observed for the patients whose target volume included CSF and vertebral bodies compared to those whose target volumes only included CSF. The thyroid, liver, heart, and esophagus are located distal to the spinal fields, and therefore dose in these organs was largely governed by their proximity anteriorly to the target volume. This distance was smaller in those patients whose spinal target volumes included the entire vertebral bodies, and thus, doses to the thyroid, liver, heart, and esophagus were higher for these patients compared to those whose target volumes only included CSF. Of these organs, the heart, which was the most anterior, had the lowest mean dose, whereas the esophagus, which was the most proximal, had the highest mean dose.

**Table 3:** The minimum, maximum, and mean dose values for the population (N = 18) in specific organs of interest are listed along with the corresponding standard deviations (SD)

		**Dose**	**SD**	**SD/Dose**	**Dose**	**SD**	**SD/Dose**	**Dose**	**SD**	**SD/Dose**
		**[cGy (RBE)]**		**(%)**	**[cGy (RBE)]**		**(%)**	**[cGy (RBE)]**		**(%)**
**N**			**18**		**4**			**14**		
**Target Volume**			**All**		**CSF space**			**CSF space and VB**		
Spinal Cord	Min	2225.3	92.3	4	2204.5	138.3	6	2231.3	73.1	3
	Max	2576.2	52.9	2	2633.5	51.3	2	2559.9	40.5	2
	Mean	2422.3	21.8	1	2436.6	11.5	0	2418.3	22.4	1
Brain	Min	2070.7	190.2	9	2182.9	145.3	7	2038.7	189.4	9
	Max	2590.3	85.9	3	2649.5	128.6	5	2573.4	59.1	2
	Mean	2419.0	25.9	1	2410.8	10.1	0	2421.3	28.5	1
Eyes	Min	379.7	302.2	80	310.0	210.8	68	399.7	320.8	80
	Max	2404.9	65.1	3	2439.0	113.3	5	2395.1	36.8	2
	Mean	1623.0	256.9	16	1533.3	186.1	12	1648.7	268.1	16
Lenses	Min	713.5	382.7	54	640.6	406.6	63	734.3	373.6	51
	Max	1602.1	241.5	15	1622.9	177.1	11	1596.1	256.6	16
	Mean	1125.9	312.4	28	1085.9	311.3	29	1137.3	311.8	27
Kidneys	Min	0.0	0.0	-	0.0	0.0		0.0	0.0	-
	Max	2153.5	318.1	15	1773.1	445.2	25	2262.2	142.3	6
	Mean	151.7	107.8	71	22.9	9.6	42	188.5	93.9	50
Lungs	Min	0.0	0.0	-	0.0	0.0	-	0.0	0.0	-
	Max	2446.3	103.4	4	2318.2	119.0	5	2483.0	60.6	2
	Mean	175.9	89.8	51	74.8	17.9	24	204.8	80.7	39
Thyroid	Min	0.0	0.0	-	0.0	0.0	-	0.0	0.0	
	Max	244.1	344.1	141	72.7	88.9	122	293.1	373.0	127
	Mean	4.9	10.2	208	0.4	0.5	125	6.2	11.2	181
Liver	Min	0.0	0.0	-	0.0	0.0	-	0.0	0.0	-
	Max	2078.0	456.5	22	1435.0	408.4	28	2261.5	262.1	12
	Mean	16.2	11.1	69	2.7	1.9	70	20.1	9.4	47
Heart	Min	0.0	0.0	-	0.0	0.0	-	0.0	0.0	-
	Max	1470.0	803.6	55	127.1	126.9	100	1853.7	404.0	22
	Mean	18.2	17.0	93	0.2	0.1	50	23.3	15.9	68
Esophagus	Min	0.1	0.4	-	0.0	0.0	-	0.2	0.4	-
	Max	1825.8	864.1	47	300.0	171.0	57	2261.8	31.1	1
	Mean	371.8	338.9	91	3.3	2.4	73	477.1	312.7	66

Also listed is the ratio of each SD to its respective dose value. Each organ was contoured in its entirety. In addition these data are presented separately for age specific target volumes, i.e., CSF space (including index 13, 15, 17, and 18) or CSF space and vertebral bodies (VB) (all remaining indices).

## Discussion

In this study, we presented a standardized methodology for treatment planning for passively-scattered proton CSI. To evaluate our planning methodology, we reviewed individual treatment plans and organ-specific population-averaged DVHs (for the 18 patients) to quantitatively evaluate the consistency of plan quality across the population. Furthermore, we examined differences in normal tissue doses related to age specific target volumes by separately evaluating dosimetric parameters for the entire population and the two target volume subgroups. We found that our standardized treatment planning method yielded plans that were dosimetrically of high quality (i.e., generally satisfied the dosimetric criteria in the Methods section) and dosimetrically consistent across the population of 18 patients. For all patients, good normal tissue sparing was achieved, but was better in the older patients whose target volumes only included CSF compared to the younger patients whose target volumes included the CSF and entire vertebral bodies.

In our evaluation of the planning technique, we focused on comparing dosimetric information for the population in ten organs: lungs, liver, heart, kidneys, spinal cord, brain, eyes, lenses, esophagus, and thyroid. The spinal cord, lenses, eyes, and thyroid were specifically identified in the planning technique for optimization. Because the spinal cord is located in the target volume, the goal of the planning method was to create a homogeneous dose across the spinal cord without hot or cold spots (dose >105% or <95% of the prescribed dose, respectively). Mean dose across the population of patients was 2519.8 cGy (RBE) (107.6%) with a 1% SD, indicating high consistency in the planning technique coverage, but because the mean dose was greater than 5% above the prescribed dose, the dosimetric analysis revealed a potential trend toward plans with a higher overall normalization. In the case of the eyes, the SD was 15%. Thus, there was relatively low variation across the population even with patient-specific edits. In the case of the thyroid, SD was high (141%), suggesting a potential need for improved standardization of the optimization technique. However, because the mean organ dose was relatively low (4.9 cGy (RBE)), the high SD may be a reflection of large variations of low dose. Moreover an alternative method for reducing dose to the thyroid that maintains the prescribed dose to the spinal cord is not known at this time.

One way that our proton CSI treatment technique could potentially be improved is through modifications to our patient immobilization and set-up. While the immobilization technique used at our institution for proton CSI achieves reproducible set-ups, it also results in increased lateral penumbra of the proton treatment fields. Specifically the 10 cm thick foam slab that is used to prevent the cranial fields from intercepting the treatment couch creates a gap between the patient and the treatment couch which results in increased lateral penumbra. The use of the foam slab was originally implemented several years ago when we used a relatively dense table-top insert that was placed on the treatment couch to facilitate indexing immobilization devices (MedTec, Kalona, IA, USA). At the time the density of the table-top insert was not a concern because we were only treating prostate cancer using lateral fields that did not intercept it. Later when faced with the first proton CSI case at our institution, we were concerned about the obliquely angled cranial fields traversing the high density indexed table-top insert. Eventually, though minor modifications to the couches top extension, we were able to index patients directly to the couch top extension and the indexing table top insert was abandoned. Subsequently, we abandoned the use of compensators for the cranial fields. With the removal of the indexing table-top insert, the removal of the compensator, and the low density nature of the t couch top extensions, it is possible that elevating of the patients with the 10 cm thick foam slab is no longer required. Reducing the thickness or possible eliminating the foam slab all together may reduce the penumbra somewhat. This would be particularly advantageous for reducing dose to the eyes and lenses. However, moving forward, we will need to carefully consider changes to patient set-up and immobilization prior to implementing any changes. Nonetheless, our experience may be useful for other institutions as they select their treatment couches and immobilization devices; table tops should be low density and allow immobilization devices to be easily indexed. Also, gaps between the treatment couch and patient should be minimized where possible.

While there is a great need for studies which compare proton treatments between institutions and with photon therapy, to date there are only a few reports in the literature that compare photon CSI and passively scattered proton CSI. Furthermore, it is difficult to fully interpret the results of these studies because none have provided a detailed description of the proton planning techniques that were used. In particular, Yuh et al. [10] reported on the toxicity of 3 pediatric patients (3–4 years of age) after CSI with protons, St Clair et al. [7] performed planning study of the comparative normal tissue sparing following conventional photon CSI, IMRT, and passively scattered proton CSI for 1 patient (aged 43 months), Yoon et al. [9] performed a dosimetric comparison of CSI using tomotherapy vs. passively scattered protons for 10 patients (mean age 7 years), Miralbell et al. [38] compared risk of second cancers following proton, conventional photon therapy, and IMRT in a 3 year old boy for spinal irradiation (cranial fields not considered), Newhauser et al. [39] compared the risk of second cancers from CSI using passively scattered proton, intensity modulated proton, conventional photon, and IMRT in a 3 year old boy, Taddei et al. [8] considered the risk of second cancers following CSI with passively scattered protons in a male patient (age 10) versus a female patient (age 9). The proton treatment planning techniques that were used in several of those studies (Table 4) indicate that the methodology for passively scattered proton CSI has varied considerably between studies. Specifically, reported methodologies vary in almost all regards from the orientation of the cranial fields to the definition of the uncertainty margins. Of particular note is that there is no specific mention of how the plans were optimized for patient-specific anatomies, i.e., there is little agreement for uncertainty margins used in the different studies, and there is no explanation of how uncertainty margins were selected. Thus, there is a substantial potential for differences in dosimetric results between studies, and this makes direct comparison of the dosimetric results difficult. Nonetheless our dosimetric results are in reasonably good agreement with previous investigations of proton CSI, including [7-10,32,38,39].

**Table 4:** Planning parameters specified in current treatment planning studies for passively scattered proton CSI

**Planning Parameters**		**Proton Planning Studies in the Literature**			
	**Yuh et al. **[10]	**St. Clair et al. **[7]	**Yoon et al. **[9]	**Miralbell et al. **[38]	**Taddei et al. **[8]
Number of Patients	3	1	10	1	2
Patient Age (y)	3 − 4	3.6	2 − 17	3	9,10
Cranial Field Orientation	RAO, LAO	RL, LL	RL, LL	None	RAO, LAO
Spinal Fields: Number, Orientation	1 − 3, PA	1, PA	1 − 3, PA	1	2, PA
Optimization	"restrict to disease site"	None stated	Proton plans re-normalized for comparison with Tomotherapy plans	constraint to achieve uniform dose to whole spinal theca	None stated
Uncertainty Margins (cm) *(with respect to clinical target)*	Not given	0.3 (Penumbra for 160 MeV protons)	Proximal: 0.2 Distal: 0.2 Lateral: 1 Smear: 0.3	0.5 (symmetric around CTV)	Not given

Abbreviations in this table: right anterior oblique (RAO), left anterior oblique (LAO), right lateral (RL), left lateral (LL), posterior-to-anterior (PA).

^*^Newhauser et al. [39] used risk contributed by infield protons reported by Miralbell et al. and added risk contributed by stray neutrons based in phantom Monte Carlo simulations of proton CSI fields (PA cranial field, PA upper spine field, and PA lower spine field).

The problem of inter-institutional variations in planning for proton CSI, including variability in target volume definitions, specifically with respect to uncertainty margins and dose constraints has been noted before [5]. Nonetheless, proton therapy is in evolution, and as the evolution progresses, the need for standardized, or at minimum documented and reproducible, planning methods increases [5]. A standardized methodology is clearly essential for robust dosimetric comparisons between centers and treatment modalities, and such comparisons are integral to progress in the area of randomized trials. In the interim, there is potential for comparative *in silico* studies, especially for populations (such as pediatric populations) too small for randomized trials. However, even *in silico* trials require a standardized planning methodology because dose distributions across tumor and normal tissues are compared, and from these, conclusions are drawn about the general effects of proton therapy relative to photon therapy as in the studies of Yoon et al. [9] and St. Clair et al. [7]). Nonetheless, progress is being made toward actual randomized trials. For example, in a recent work, Howell et al. [31] performed a dosimetric comparison using an actual patient population and the standardized treatment planning methodology outlined here, and they found that proton CSI improved normal tissue sparing while also providing more homogeneous target coverage than photon CSI. Thus, the potential for similar studies is growing, and with increased standardization, the evidence from these studies is becoming increasingly robust.

## Conclusions

This study described and evaluated a standardized method for proton treatment planning for CSI. Overall, the standardized planning methodology yielded consistently high quality treatment plans and perhaps most importantly, it did so for an anatomically diverse patient population without creating outliers. Finally, the planning methodology described here can be used to provide guidance to other proton centers as they implement CSI.

## Consent

Written informed consent was obtained from the patient for publication of this report and any accompanying images.

## Competing interests

The authors declare that they have no competing interests.

## Authors’ contributions

AG created the proton treatment plans, performed data analysis, and drafted the manuscript. WN supervised retrospective collection of patient data, reviewed data analyses, and edited the manuscript. RA provided guidance on clinical aspects of the manuscript, particularly those pertaining to patient set-up and field definition, and edited the manuscript. AM provided guidance on all clinical aspects of this manuscript, reviewed and approved each proton treatment plan, and edited the manuscript. KH reviewed/edited the manuscript and participated in various aspects of data analysis including writing an excel macro to process the Eclipse DVHs and extract specified dosimetric parameters. RH designed this study, provided scientific leadership to the research team, mentored AG in the writing the manuscript, reviewed data analyses, reviewed/edited all drafts of the manuscript, and prepared the revised manuscript and responded to reviewer comments. All authors read and approved the final manuscript.
